# Effects of a Financial Incentive on Health Researchers’ Response to an Online Survey: a Randomized Controlled Trial

**DOI:** 10.2196/jmir.1251

**Published:** 2010-05-10

**Authors:** Paul M Wilson, Mark Petticrew, Mike Calnan, Irwin Nazareth

**Affiliations:** ^4^MRC General Practice Research FrameworkUniversity College LondonLondonUnited Kingdom; ^3^School of Social PolicySociology and Social Research, University of KentCanterburyUnited Kingdom; ^2^Public and Environmental Health Research UnitLondon School of Hygiene and Tropical MedicineLondonUnited Kingdom; ^1^Centre for Reviews and DisseminationUniversity of YorkYorkUnited Kingdom

**Keywords:** Questionnaires, Electronic Mail, Randomized Controlled Trial, Reminder Systems, reward

## Abstract

**Background:**

Nonresponse to questionnaires can affect the validity of surveys and introduce bias. Offering financial incentives can increase response rates to postal questionnaires, but the effect of financial incentives on response rates to online surveys is less clear.

**Objective:**

As part of a survey, we aimed to test whether knowledge of a financial incentive would increase the response rate to an online questionnaire.

**Methods:**

A randomized controlled trial of 485 UK-based principal investigators of publicly funded health services and population health research. Participants were contacted by email and invited to complete an online questionnaire via an embedded URL. Participants were randomly allocated to groups with either “knowledge of” or “no knowledge of” a financial incentive (£10 Amazon gift voucher) to be provided on completion of the survey. At the end of the study, gift vouchers were given to all participants who completed the questionnaire regardless of initial randomization status. Four reminder emails (sent from the same email address as the initial invitation) were sent out to nonrespondents at one, two, three, and four weeks; a fifth postal reminder was also undertaken. The primary outcome measure for the trial was the response rate one week after the second reminder. Response rate was also measured at the end of weeks one, two, three, four, and five, and after a postal reminder was sent.

**Results:**

In total, 243 (50%) questionnaires were returned (232 completed, 11 in which participation was declined). One week after the second reminder, the response rate in the “knowledge” group was 27% (66/244) versus 20% (49/241) in the “no knowledge” group (χ^2^_1_ = 3.0, P = .08). The odds ratio for responding among those with knowledge of an incentive was 1.45 (95% confidence interval [CI] 0.95 - 2.21). At the third reminder, participants in the “no knowledge” group were informed about the incentive, ending the randomized element of the study. However we continued to follow up all participants, and from reminder three onwards, no significant differences were observed in the response rates of the two groups.

**Conclusions:**

Knowledge of a financial incentive did not significantly increase the response rate to an online questionnaire. Future surveys should consider including a randomized element to further test the utility of offering incentives of other types and amounts to participate in online questionnaires.

**Trial Registration:**

ISRCTN59912797; http://www.controlled-trials.com/ISRCTN59912797 (Archived by WebCite at http://www.webcitation.org/5iPPLbT7s)

## Introduction

Nonresponse to questionnaires can affect the validity of surveys and introduce bias.

The offer of financial incentives has been a widely used method to increase response rates to postal questionnaires. A Cochrane systematic review of 481 randomized controlled trials (RCTs) evaluating 110 different ways of increasing response rates to postal questionnaires in a wide range of populations found that odds of response can be doubled through the use of monetary incentives [1]. Other factors shown to increase the odds of response included a topic of interest, pre notification, follow-up contact, unconditional incentives, shorter questionnaires, provision of a second copy of the questionnaire at follow-up, mention of an obligation to respond, and university sponsorship [1].

However, this evidence base relates to postal questionnaires, and although a number of systematic reviews [1,2] and meta-analyses [3] have been conducted, the available evidence base relating to use of incentives in electronic questionnaires is less substantive. The Cochrane review included 32 RCTs that evaluated 27 different ways of increasing response rates to electronic questionnaires in a wide range of populations [1]. Although the one included RCT that evaluated monetary incentives found no significant effect, a further six RCTs found that use of other financial incentives (such as Amazon gift vouchers) doubled the odds of response. Limited evidence from social and market research also suggests that the offer of some form of monetary or financial incentive can increase the odds of a person responding and completing a web survey [3].

Theoretical frameworks have been used to explain the potential influence of incentives on response rates. Social exchange theory [4] proposes that the actions of individuals are influenced by the balance between the rewards they expect to obtain and the costs they perceive may occur as a consequence; this exchange paradigm has become a key concept in marketing [5]. A systematic review of the design and conduct of questionnaire surveys suggests that making exchange theory operational (in order to maximize response)involves minimizing the physical, mental, emotional, and economic costs of response, maximizing the tangible and intangible rewards for response, and establishing trust that those rewards will be delivered [6]. In contrast, Leverage-saliency theory [7] proposes that a potential participant’s decision to respond to a survey is influenced by the importance placed on key factors such as interest in the topic, [8] available time; the credibility of the research source, and the benefits (tangible or otherwise) the individual perceives will result from participation. The theory postulates that potential participants with a strong interest in the topic are more likely to respond; incentives can act as leverage for those potential participants for whom influencing factors (such as topic of interest) are deemed less important.

Our study was undertaken as part of a survey to assess what steps researchers in the fields of health service and population health within the United Kingdom are taking to disseminate the findings of their research. Addressing deficiencies in the dissemination and transfer of research-based knowledge into routine clinical practice is high on the policy agenda both in the United Kingdom [9-11] and internationally [12]. Research dissemination and knowledge transfer is also highly relevant to the United Kingdom applied health research community. The main funder, the National Institute for Health Research (NIHR), is seeking to maximize the impact of its £800 million investment in applied health research [13]. The NIHR has expectations that researchers will work to ensure that research is made available, can be used to support decision making, and will ultimately improve the quality and delivery of health care.

The population of interest for this survey is university-based and has high levels of Internet and email access. In addition, the major public funders of public health and health services research in the United Kingdom operate electronic online submission processes and use email as the principal mode of communication with grant holders and applicants. Given this, we decided to adopt a Web-based survey approach as it represented the most efficient and low cost mode of delivery.

However, there is some evidence that Web-based surveys can result in lower response rates (around 10%) compared with other survey modes [2,14,15]. Because of this, we decided to offer an incentive (gift vouchers from the online retailer Amazon) to participants to respond. Although a variety of incentives to increase response rates have been tested in a wide range of professional populations (including nine previous studies involving faculty members at universities [1]), to our knowledge there is no evidence based on a randomized trial relating to our specific population of interest. In addition, the Cochrane review included three randomized evaluations of Amazon gift vouchers that showed mixed effects [1]. Given this, we decided to test—using a randomized controlled trial nested within a survey—whether knowledge of a financial incentive would increase the response rate to the online questionnaire.

## Methods

### Recruitment

In July 2008, after obtaining ethical approval for the study from the University of York IRISS Ethics Committee, we contacted 10 UK programs and agencies that fund health services and public health research. The agencies were invited to provide (secure and encrypted) email contact details for UK-based principal investigators of health services and public health research completed in the last five years (2003-2008). Five agencies (the Scottish Chief Scientist Office, Economic and Social Research Council, Medical Research Council, NIHR Health Technology Assessment Programme and Wellcome Trust) responded and provided details. Principal investigator details for one non responding agency (NIHR Service Delivery and Organisation Programme) were publicly available and were obtained from their website. Two agencies (British Heart Foundation and Joseph Rowntree Foundation) indicated that they fund very little public health and health services research and so were excluded from the survey. The Department of Health Policy Research Programme and Cancer Research UK responded stating that they were unable to provide details of principal investigators.

We identified 743 principal investigators from the six funding agencies. Duplicates were removed from the list resulting in a total survey sample of 536 potential participants. Email addresses for identified principal investigators were then checked and compiled.

### Study design and randomisation

Potential participants were randomized to receive either “knowledge of” or “no knowledge of” a financial incentive—in this instance gift vouchers (from the online retailer Amazon) to the value of £10. Amazon gift vouchers (distributed via the Amazon email gift certificate facility) were sent to all participants who completed the questionnaire regardless of the study group to which they were randomized.

Random allocation of participants using computer-generated numbers was undertaken independently by a statistician at the Medical Research Council (MRC) General Practice Research Framework.

### Administration

On October 13, 2008, both groups were contacted by email (Textbox 1). Participants were told the purpose of the study and invited to complete an online questionnaire via an embedded URL. The online questionnaire was hosted on the SurveyMonkey website [16] and was based on an instrument previously used to assess the practices of intramural MRC Research Units in an earlier phase of the project. The questionnaire comprised a combination of 36 open and closed questions that could be completed in 20 to 30 minutes. The questionnaire was piloted prior to use.

**Email invitation to knowledge group**

**Subject: MRC PHSRN survey invite**

Dear Colleague,

Disseminating the Findings of Health Services and Public Health Research

We are writing to invite you to take part in a survey.

This survey aims to find out what steps public health and health services researchers working across the United Kingdom are taking to disseminate the findings of their research.

The survey is part of a three-year project funded by the MRC Population Health Sciences Research Network (Ref: PHSRN 11). The project aims to identify ways by which the uptake of publicly funded public health and health services research can be enhanced.

We very much hope that you will agree to participate and complete the questionnaire.

The questionnaire contains 36 questions and can be completed in 20-30 minutes.

Respondents who complete the full questionnaire will receive a £10 Amazon gift voucher.

Any information provided will be treated in the strictest confidence and presented on a nonattributed basis.

Click here to go to the questionnaire. http://tinyurl.com/5olpfq

Please do not circulate to other colleagues

Thank you for your cooperation.

Best wishes

Paul Wilson

On behalf of:

Mark Petticrew, London School of Hygiene and Tropical Medicine

Mike Calnan, University of Kent

Irwin Nazareth, MRC General Practice Research Framework

Paul Wilson

Centre for Reviews and Dissemination

University of York

York

YO10 5DD

t: +44 (0)1904 321073

f: +44 (0)1904 321041

e: pmw7@york.ac.uk

The email sent to the participants in the “knowledge” group stated that those who completed the online questionnaire would receive a £10 Amazon gift voucher. The study design specified that four reminder emails (sent from the same email address as the initial invitation) would be sent out to nonrespondents at one, two, three, and four weeks following the initial invitation; a fifth postal reminder would be sent to nonrespondents if the response rate was considered to be low. Participants who completed the online questionnaire were deemed to have given their consent. Questionnaires not returned by December 31, 2008, were deemed to be nonresponses.

As this RCT was nested within a larger survey, the primary concern was to maximize response rates. Given this, it was determined thatif the difference in the response rate between the two groups was such that it was likely to adversely affect the main aims of the survey, then knowledge of the incentive would be provided to the “no knowledge” group, but not before the third reminder. At the third reminder, we provided “knowledge of” the incentive to the “no knowledge” group to limit any adverse effects on total response to the survey.

A combination of IP address and questionnaire responses were used to identify multiple responses from a single participant [17]. Where multiple responses from a single participant occurred, the most recently completed questionnaire was retained for analysis. Noninvited responses from individuals not part of the study sample were excluded from the analysis.

### Analysis

The primary outcome measure for the trial was rate of response one week after the second reminder. Rate of response was also measured at the end of weeks one, two, three, four, and five, and after the postal reminder. Data were entered and analysed in SPSS version 15.0 (SPSS Inc, Chicago, IL, USA). We compared the response rates in each group using the chi-square statistic.

## Results

**Figure 1:**
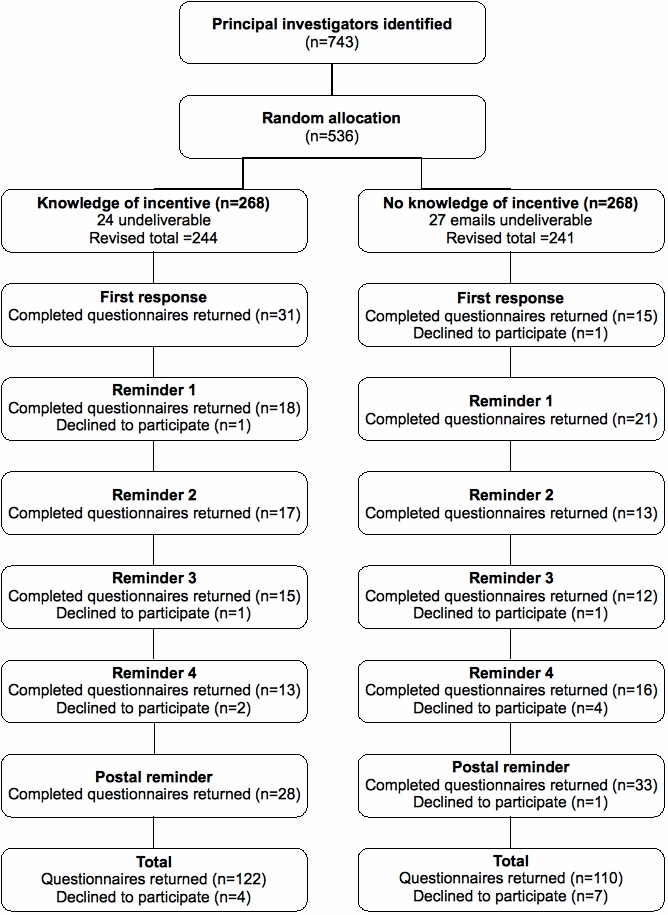
Flow chart of “knowledge of” versus “no knowledge of” financial incentive

Of the 536 identified email addresses, 51 were undeliverable resulting in a sample of 485. A total 243 (50%) questionnaires were returned (232 completed; 11 in which participation was declined). Figure 1 illustrates the flow of responses to the study.

As a measure of completion [17], 100% of the 232 participants who completed questionnaires answered the questions on the first page, and 95% (220/232) answered the final question. Excluded from the analyses were 4 questionnaires completed by noninvited individuals. Multiple responses were submitted by 2 participants; the most recently submitted questionnaire was included in the analyses in each case.

**Figure 2:**
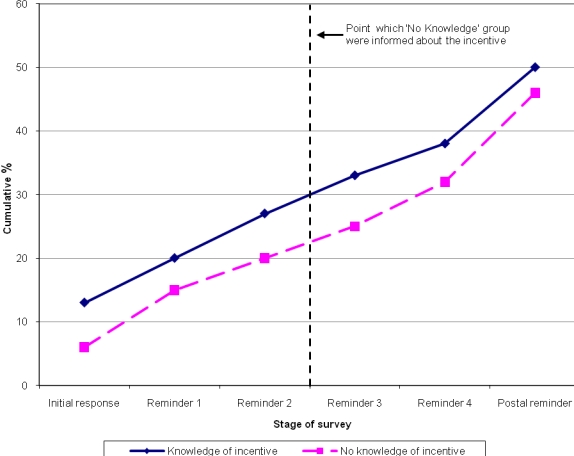
Cumulative response (%) over time by group

Table 1 shows the cumulative response rate over time by group. Figure 2 shows the cumulative percentage response over time, again by group. The primary outcome measure for the trial was rate of response one week after the second reminder. The cumulative response rate in the ”knowledge” group was 27% (66/244) versus 20% (49/241) in the “no knowledge” group. This difference was not statistically significant (χ^2^_1_=3.0, *P*=.08). The odds ratio for those with knowledge of an incentive that responded was 1.45 (95% confidence interval [CI] 0.95 - 2.21).

**Table 1:** Cumulative response over time by group

	Knowledge Group (n = 244)	No Knowledge Group^a^ (n = 241)	χ^2^ Significance
First response	31 (13%)	15 (6%)	*P*=.01
Reminder 1	49 (20%)	36 (15%)	*P*=.13
Reminder 2	66 (27%)	49 (20%)	*P*=.08
Reminder 3	81 (33%)	61 (25%)	*P*=.06
Reminder 4	94 (38%)	77 (32%)	*P*=.13
Postal Reminder	122 (50%)	110 (46%)	*P*=.33

^a^No knowledge group informed about incentive from reminder 3 onwards

At the third reminder, participants in the “no knowledge” group were informed about the incentive, ending the randomized trial nested within the survey. As this was a survey, we continued to follow up all respondents, and for transparency purposes, Table 1 presents further data on the cumulative response rates. No significant differences were observed in response rates between the two groups from reminder 3 onwards.

## Discussion

### Statement of principal findings

Knowledge of a financial incentive did not significantly improve the response rate to this online questionnaire. However, one week after the second reminder—the point before the “no knowledge” group were informed about the incentive—a difference of 7% was apparent.

### Comparison with other studies

In terms of overall response, our rate of 50% compares favourably with those reported for other Web surveys. For example, in a review of comparisons of Web survey versus other survey modes, only 6 of 45 Web surveys managed to obtain a response rate higher than 50% [14]. In a second meta-analysis, which reported an 11% difference in response rates in favor of postal over Web modes, only 10 of 39 comparisons obtained a Web survey response rate higher than 50% [15]. Previous randomized evaluations of our choice of incentive (an Amazon gift voucher) [1] have shown mixed effects in different populations and settings. However, of three previous studies similar to ours included in the Cochrane review, researchers compared the effects of: a $5 cash incentive versus a $5 gift voucher [19]; no incentive versus entry into a lottery for $50, $100, $150, or $200 gift vouchers [20]; and unconditional $15 or $25 gift vouchers versus $15 or $25 gift vouchers conditional on completion of the survey [21].

### Strengths and limitations of study

In developing our survey, we adhered to recommendations for the design of email questionnaires [18]. These included deriving an appropriate sample, using an embedded URL, using incentives, and sending the request for information from a recognized academic source. One recommendation beyond our control was that the research be perceived to be relevant to the population surveyed. As stated above, there is renewed emphasis on increasing the uptake and transfer of publicly funded research into policy and practice, and those responding indicated that dissemination of the results of research was highly relevant to their work. However, we had no way of knowing beforehand whether the topic or goal would be deemed relevant or of interest by those we surveyed.

In our study, we utilized a 36-item questionnaire and stated that it would take participants up to 30 minutes to complete. Shorter postal questionnaires are associated with increased response rates [1]. It may be that the perceived return (£10) for time invested in completing the 36 items was deemed inadequate compensation by some participants, especially if considered in relation to their incomes as professional researchers. We do not know whether an increase in the financial incentive relative to participant income would have made any difference in this instance. Another consideration relates to the nature of the incentive offered. Receipt of the gift voucher was dependent on the participant completing the questionnaire. There is evidence that response rates can be higher when an incentive is given up front unconditionally rather than given conditional on completion [1]. The use of unconditional versus conditional incentives merits further investigation.

In this study, members of the population of interest have high levels of Internet and email access. Yet, around a fifth of all returned completed questionnaires were paper copies that had been mailed out as part of the postal reminder. This decision to adopt a mixed mode approach in the event of a low response rate appears sensible in light of feedback from two of the respondents. They indicated that they found it hard to find the time to respond to Web surveys, and as they were often out of the office, it was easier to complete a survey that used a paper-and-pen format. Although we recognize that our experience relates to a very specific population and suggest some caution in generalizing these findings to other populations, designers of future Web surveys may wish to consider using this mixed mode approach.

This randomized study was undertaken as part of a wider survey to assess what steps public health and health services researchers working across the United Kingdom are taking to disseminate the findings of their research. This nesting approach offered a cheap and efficient method of adding to our knowledge of the utility of different survey modes. However, undertaking such an approach was not without potential challenges. Normally in randomized studies, one would compare an intervention against standard practice when the outcome is unknown. But in this instance our primary concern was to maximize response rates to the wider survey. In doing so it was possible we limited the duration of the intervention making it difficult to determine what the true effect of the incentive would have been over a longer time period. Future web surveys should consider nesting a randomized element to further test the utility of incentives but should also consider whether the time frame for response is adequate to determine the true effect.

### Conclusions

Our trial can help researchers planning future Web-based surveys. It would appear that immediate responses within two weeks of initial contact to a Web-based survey might be improved by the offer of a small financial incentive. Hence, we would recommend small financial incentives to those researchers requiring quick responses to Web-based questionnaires. However our findings suggest that this effect may dissipate over time. Researchers should consider that even in specific populations with high levels of access to the Internet, there might be advantages in using mixed methods (ie, use of both web and paper questionnaires) in terms of participant preferences and in increasing response rates.
